# Congenital absence of touch does not preclude normal cognitive and socioemotional development

**DOI:** 10.21203/rs.3.rs-4791322/v1

**Published:** 2024-08-07

**Authors:** Peggy Mason, Anthony Reder, Maureen Lacy, Jayant Pinto

**Affiliations:** The University of Chicago; The University of Chicago; The University of Chicago; The University of Chicago

**Keywords:** Hereditary Sensory Autonomic Neuropathy, attachment theory, somatosensation, embodiment, gustation, disability

## Abstract

Attachment theory holds that development of normal affective and social behavior requires physical contact between infant and caregiver. The elevation of touch to paramount importance has gone unchallenged because, prior to the present study, no individual with a congenital lack of somatosensation has been reported, much less studied for psychosocial development. Here we describe Kim, who since birth, has been unable to perceive touch, temperature changes, or pain on the body surface. Despite her inability to sense physical contact, Kim has above-average intelligence. She functions normally in social situations with a variety of people, recognizing emotions in herself and others and demonstrating appropriate affect. Kim experiences anxiety that appears grounded in realistic fears and uncertainties particular to her somatic insensitivity, thus serving as adaptive vigilance in reaction to an abnormal sensorium. Her normal socioemotional development, evident from an early age, likely resulted from Kim being able to appreciate her parents’ loving care through gaze, movement, and hearing. In sum, Kim upends the idea of touch as critical to developing a sense of self, secure attachment, and family bonds.

## Introduction

Picture a child running full speed to her father, wrapping her arms around his legs. The father leans down, gives her a hug and a pat. In the next instant, she is off again, beaming as she ventures out to play with other children, familiar and unfamiliar alike. This scene, repeated at family outings the world over, forms the essence of attachment theory which holds that making ***physical contact*** with a caregiver serves as “the primary signal to infants that they are … safe and secure” (McGlone et al 2014). A secure attachment in turn “facilitates healthy self-reliance,” fosters exploration, and ultimately influences how the grown infant “organizes behavior toward … the environment, both animate and inanimate” (Ainsworth, 1979).

Central to successful attachment is the touch of physical contact. As articulated by Ruth Feldman (2011), “Maternal touch... serves as the bedrock of the individual’s future capacity to provide love and nourishment to future attachment relationships.” The positive impact of skin-to-skin contact between mother and neonate exemplifies the idea that touch triggers attachment (Bigelow and Power 2020, Feldman et al 2002, Rheinheimer et al 2022). Experiments in non-human animals from worms to rats and non-human primates show that the benefits of touch on physical, behavioral, and social outcomes are shared widely across phylogeny (Ardiel and Rankin 2010, Feldman 2011, Field 2014, Stack and Jean 2011, Harlow 1958, 1959; Suomi 1984).

Experiments in rats and monkeys evidence the role of touch *per se*. For example, the reduced growth and heightened stress reactivity produced by separating rat pups from their mother are almost completely reversed by brushing stimulation (intended to mimic the mother’s licking) but unchanged by either vestibular or kinesthetic stimulation (Pauk et al 1986). Stroking with a paint brush rescues many of the adverse consequences of physical isolation, suggesting that stimulation of mechanosensitive afferents is critical to normal development (Ardiel and Rankin 2010, Gonzalez et al 2001). Isolating baby macaques from physical contact with the mother and peers results in repetitive stereotypies, deficits in exploration and social play, and poor outcomes for the offspring of isolated females due to poor mothering (Harlow 1959, Suomi 1984). These adverse consequences are not prevented by auditory and visual access to other monkeys. Reminiscent of the strong attachment that babies show for soft cloth, baby macaque monkeys cling to a terry cloth mother-substitute without milk rather than a chicken-wire-substitute with milk, dramatically demonstrating mammalian offspring’s preference for soft touch even over nourishment (Harlow 1958, 1959).

The touch engaged by physical contact and implicated in the development of secure attachment may primarily or even exclusively depend on unmyelinated, touch-sensitive afferents from hairy skin (CT afferents; Cascio et al 2019, McGlone et al 2014). Yet, experiments examining human psychosocial development without touch have been impossible to date because of technical – somatosensation is not gated as are vision and hearing – and obvious ethical constraints. Here we take advantage of a unique individual with a congenital sensory neuropathy to ask whether experiencing touch is critical to social and affective development. We describe a healthy, 45-year-old, disabled woman who was diagnosed with Hereditary Sensory and Autonomic Neuropathy Type II at the age of thirty-one months (Axelrod and Gold-von Simpson 2007). We identify her as Kim in accordance with her expressed preference (see Supplementary Material).

After establishing that Kim is and always has been insensitive to cutaneous somatosensory and gustatory stimulation, we report the results of a neuropsychological evaluation assessing her cognitive and socioemotional state.

## Materials and methods

### Informed consent

All procedures took place at the University of Chicago and were approved by the University of Chicago Institutional Review Board. An independent bioethicist discussed the overall study with Kim and obtained informed consent.

### Medical records

Medical records from Drs. Margaret Hack (Rainbow Babies & Children’s Hospital, 1979–80), Peter Dyck (Mayo, 1981), and Felicia Axelrod (NYU, 1979, 2006) were made available to the authors and are referenced. Additional history was garnered from Kim and her mother.

### Taste testing

To test taste, *Burghart* taste strips, a well-studied standardized psychometric test of gustatory perception, were used (Landis et al 2009). Adapting this test to Kim, taste strips were handled by an investigator (see Movie 1).

### Smell testing

The University of Pennsylvania Smell Identification Test^™^ (UPSIT) from *Sensonics* (Haddon Heights, NJ, USA) along with marker-based tests for olfactory threshold, discrimination, and identification from *Burghart* were used (Hummel et al 1997, Doty 1995, Doty et al 1984, Hummel et al 2007).

### Neuropsychological testing

Kim was examined twice at age 44 with two-day visits separated by six months. A licensed clinical neuropsychologist conducted the clinical interviews. Neuropsychological technicians administered testing and informally interacted with Kim. These technicians were deliberately chosen to assess Kim’s ability to relate to people who are diverse with respect to age, sex, and personality.

## Results

### Brief history

After an uncomplicated pregnancy, Kim was born 8 weeks early with a normal birth weight for her gestational age. At birth, she was termed “floppy.” She did not cry or otherwise show discomfort in response to somatic stimuli including heel stick and blood draws. Corneal breakdowns were noted within her first year and have continued periodically to date. Kim did not show suck, gag, or swallow reflexes. Neonatal course was complicated by “severe hypotonia” and a failure to suck. Kim was fed by nasogastric tube until roughly 6 months when she learned to swallow. Kim has never self-mutilated, likely secondary to her not placing digits in her mouth.

Examinations before the age of 3 revealed decreased fungiform papillae on the tongue “normal filiform papillae and some fungiform, the latter were rudimentary.” She had absent deep tendon reflexes; normal compound motor action potentials but completely absent sensory nerve action potentials; an absence of sensory-evoked cortical potentials; an abnormal intradermal histamine response; normal sweating; and a sural nerve biopsy showed empty nerve sheaths at the light microscopic level.

At age 31 months, an evaluation at the Mayo Clinic revealed that Kim had a good vocabulary and spoke in complete sentences. She was reading by 3 years old. Once in school, Kim excelled academically. She was placed in gifted classes in grades 2–8 and in honors classes during high school. She earned a bachelor’s degree in Business Administration and a JD, the postgraduate US law degree. Kim passed the bar exam on her first attempt. She is currently employed in legal research.

Kim does not stand or walk independently and uses a wheelchair for mobility.

Kim was “socially responsive” when examined at NYU at 7 months of age. Today, she is socially active within her family and social group.

As a teenager, Kim began to have bouts of vomiting related to anxiety. With buspirone, taken on and off as needed since age 16, these bouts of vomiting have resolved.

Today, Kim is in good health. She has one sibling, an unaffected sister.

### Sensorium

Vision, hearing, and vestibular function were all normal.

#### Taste

Kim clearly and consistently pronounced liking or not liking the *taste* of particular foods. Yet, at age 35, she reported not knowing the meaning of taste words including *sweet* and *salty.* This linguistic lacuna suggested that Kim lacks gustatory perception while using olfaction and vision to achieve a sense of *flavor*, the colloquial meaning of *taste*.

To test gustatory perception, we used taste strips for salt, sweet, sour and bitter. For each taste, four concentrations were presented, starting with the lowest concentration. The order of tastes was random at each concentration level. Up to three trials with blank taste strips were intermingled among the presentations. Each strip was placed on Kim’s tongue and moved around before being removed (see Movie 1). Kim then reported her choice. Between successive strips, she drank a swig of water.

Test protocol calls for participants to choose between the four tastes and *no taste*. However, since taste words hold no meaning for Kim, we initially performed the test (age 38) with a sixth possible choice that was positive for detection but unspecified as to which taste: *taste*.

Kim chose *taste* for 10 of the 16 taste strips, distributed across the four concentrations. She identified the one blank strip presented to her as *no taste*.Kim offered only one named taste, saying “ew weird taste, maybe sour” to the highest concentration of sour. She also reported reactions of unusual valence. To the lowest concentration of sweet, Kim responded, “definitely a taste” and then added with a grimace, “eww… not a good one either” (Movie 1). She then immediately asked for water. Kim’s perceptions were not consistently related to intensity. For example, Kim responded *no taste* for the highest two concentrations of salty but *taste* for the lowest two concentrations.

Two years later, when Kim was 40, we repeated the test, asking Kim to choose from the standard five responses. Kim answered correctly for one of the 16 tastant strips (*sweet* for the lowest concentration of sweet) and for one of three blank strips presented. Beyond this, Kim’s answers were inconsistent. For example, she answered *sour* to the highest concentration of sweet, the second highest concentration of bitter, and to one of the blank strips. Kim’s responses placed her in the *ageusic* category (Landis et al 2009).

#### Smell

To get an overall sense of Kim’s ability to perceive common odors, we administered the UPSIT from *Sensonics*. This consists of 40 scratch-and-sniff odors that the participant identifies as one of four listed choices. Kim scored 17/40 which is above chance but in the anosmic range for adult women (Doty 1995, Doty et al 1984). In a second independent odorant-naming task (Sniffin’ Sticks), Kim correctly identified 12/16 odors from a list of four possibilities which matched the 10^th^ percentile for asymptomatic women in her age range (Hummel et al 1997).

To interrogate Kim’s olfactory abilities independent of nominal semantics, we used “Sniffin’ Sticks” to examine olfactory threshold and discrimination (Hummel et al 1997). Kim’s detection threshold for n-butanol was normal (Hummel et al 2007). However, Kim scored below the 5^th^ percentile for her age and sex in distinguishing which of three odors differed (Hummel et al 2007). Kim’s composite score (TDI) was between the 5^th^ and 10^th^ percentiles, placing her in the hyposmic range (Hummel et al 2007).

#### Somatosensation by history and exam

Histamine evoked a wheal but no flare at ages 5 weeks and 7 months. After an exam at 7 months, she was described as having “the most complete sensory loss imaginable.” At age 31 months, no sural nerve action potential could be detected and a sural nerve biopsy revealed no myelinated fibers. At 26 years, Kim was completely insensitive to warm, cold, and vibratory stimuli examined using the Medoc system.

At age 38, Kim was examined by the authors. With her eyes closed, Kim did not respond to touch with a fine filament, sharp point, or cold probe applied anywhere on the face or body. She showed no awareness of the position of her distal limbs, toes, or fingers. She did not feel a vibrating 125 Hz tuning fork on her limbs. However, when a vibrating tuning fork was applied progressively nearer the spine, the site of a scoliosis-correcting C7-to-pelvis-rod, she was increasingly sensitive from about T10 upwards, reporting that she sensed the stimulus “in her ears.”

Beyond our examination, life events illustrate Kim’s cutaneous sensory blindness. Clinical notes accompanying the sural nerve biopsy reported, “The child did not react to the skin incision or to the transection of the nerve fibers.” In a middle school home economics class, Kim held a pizza pan just removed from the oven without discomfort. Kim only realized that damage had occurred hours later when she saw extensive blistering on her hand.

Throughout her life, Kim has periodically experienced corneal ulcerations, all without discomfort. In her mid-twenties, Kim did not know that a bottom incisor (after being tapped with a water bottle) had fallen out until she saw blood coming from her mouth. Kim regularly finds cuts and abrasions on herself for which she is never able to recall a causative incident.

Beyond an insensitivity to noxious stimuli, Kim has indirectly evidenced a lack of touch throughout her life. As a baby she showed no interest in blankets, pillows, or other soft objects. She never sucked her thumb. Explaining her desire to not be in the dark, Kim said, “Because I don’t have any sensation to the world. I don’t have any sense of touch or anything. My vision is how I pretty much orient myself to the world. So pretty much if you take my vision away, it’s very disorienting. I can’t feel where I am and things like that.”

### Neuropsychological testing

Formalized cognitive assessment revealed above-average to average abilities despite motor limitations hindering optimal performance on timed motor-based measures (e.g., block design). Specifically, Kim demonstrated above-average intelligence, high-average learning of verbal material, and average to high-average immediate and delayed recall and recognition memory skills. She performed in the above-average range for immediate and delayed recall of visual material. Assessment of language skills revealed exceptionally high word knowledge, verbal concept formation, and applied lexical knowledge; with average speeded phonemic and semantic fluency skills. Her performance on confrontation naming was in the exceptionally low range. Kim missed 3 words related to cooking and food but recalled all with cues. Of note, Kim doesn’t cook for herself. She performed in the average range for visuoperceptual reasoning, visual integration, and organizing visual stimuli tasks. Across executive tasks she displayed above-average sustained attention, working memory, mental flexibility, abstract reasoning, and problem-solving skills.

On formal assessment via completion of the Autism Diagnostic Observation Schedule (ADOS) by a trained psychologist, an Autism Spectrum Disorder (ASD) diagnosis was not supported. Interviews and responses to formal measures assessing ASD symptomatology completed by her sister and her mother again was not indicative of ASD. They each denied early social functioning deficits, emotional dysregulation, or rigidity.

Direct observations regarding social comportment, conversational exchanges, and mental flexibility with examiners further buttressed the above conclusion. Specifically, Kim demonstrated appropriate eye contact, vocal tone, prosody, and gesturing. She was pleasant, cooperative, and mood-congruent thoughout the assessment sessions. Kim appeared motivated and attentive to task demands. Rapport was easily established and maintained with technicians and psychologists. Her facial expressions were congruent with her affective state and thought context. She appropriately provided information to the examiners regarding her thoughts, feelings, and experiences. As appropriate, she inquired about the examiner’s thoughts, feelings, and experiences. She demonstrated effective verbal social overtures. She appropriately provided goal directed responses void of delusions or grandiosity. Upon inquiry, Kim identified and described her emotions with ease.

Kim reported feeling “socially behind” her peers in grammar school and having challenges making friends because “typical kids... did not want to associate” with her. She spoke insightfully of the stigma around her physical presentation. Upon entering high school, she reported her social challenges mitigated due to finding a more mature group of friends. She continues to retain friends from high school and college to this day. Kim reported experiencing love, intimacy, disappointment, anger, conflict, and resolution in her relationships. As she reported these experiences, Kim showed congruent facial expressions (e.g. smiling, crying) and body language (e.g. stillness, prolonged eye contact).

Kim’s emotional descriptions were notably cognitive and motor based without a sensory component such as a racing heart or sweaty hands. For example, Kim stated that, when anxious, her mind races and she feels a constant drive to swallow and tighten her muscles. When angry, she feels “a war in [her] head.” Overall, observations revealed intact socioemotional and age-appropriate social skills.

On a self-report stigma questionnaire, Kim’s responses indicated an elevated level of stigma. Kim reported being treated with less respect than other people. She perceived others acting afraid of her at a frequency of a few times a month. She experienced being treated with less courtesy than others. Notably, Kim reported experiencing others as showing that they think her to be intellectually challenged, apparently based on her appearance. In one memorable occasion when Kim was in gifted classes in middle school, a physician asked Kim’s mother (rather than Kim who was present) if she went to school and if she could read. Today, health care workers often talk to someone accompanying Kim rather than to Kim.

On a self-report personality inventory, inspection of the validity profile indicated a consistent and forthright response to questions. The clinical profile was void of any indication of the presence of clinical psychopathology; yet, Kim’s responses resulted in an elevated Anxiety subscale score indicative of a clinical level of anxiety in the mild range of severity. This composite assessment derived almost entirely from cognitive and affective components; Kim selected few items in the physiological component that concerns sensory perceptions such as damp palms or pounding heart. The clinical profile also suggested a strong need for approval and acceptance. Along with anxiety, Kim’s responses resulted in a score suggestive of elevated aggression. As noted above, Kim often encounters stigma around her physical disability with assumptions around her intellect. She spoke of this as frustrating and demeaning, which along with heightened anxiety, may contribute to the elevation in aggression.

Kim rarely feels sensations that arise from within. When they do occur, she perceives them as unpleasant and potentially dangerous. The potential for danger is epitomized by her fear of contracting an entirely treatable and yet, if not treated, fatal condition such as a burst appendix that she could not feel due to her “disability.” With this cognitive concern, rare internal sensations easily divert Kim into a state of anxiety.

In terms of functional impact, Kim directs aides to help physically complete many basic and instrumental activities of daily living (ADLs, bathing, dressing, toileting, getting in and out of bed, eating; IADLs, preparing meals, housekeeping, transportation, taking medications). For example, she picks out her clothes but relies on an aide to put them on her. She chooses her meals and feeds herself food (using a mirror and specialized spoon) that someone else has prepared and cut up. She determines when to void and reaches the toilet with the assistance of an aide. She voids on her own and then an aide wipes her. Kim keeps her prescriptions up to date and prompts an aide to administer medications. Since college, Kim has organized coverage for her own care by hiring, scheduling, and arranging payment for aides. In this way, beyond being directly responsible for quotidian activities such as using a phone and shopping online, Kim is indirectly responsible for daily tasks.

## Discussion

Despite a complete insensitivity to cutaneous stimulation, Kim exhibits above-average intelligence and exhibits no social or emotional pathologies. These findings show that tactile, thermal, and other cutaneous somatosensation along with taste perception, also absent in Kim, are not required for normal cognitive, emotional, and social development.

### Kim lacks somatosensation and gustation

The conscious discrimination of the *where*, *when*, and *what* of cutaneous stimuli constitute the typical meaning of tactile perception. Such discriminative information is carried by myelinated afferents, whereas CT afferents support positive affective reactions to mechanical stimulation that are poorly localized and loosely anchored in time (McGlone et al 2014). CT afferent activation by slow stroking of the hairy skin has been termed *pleasant* or *social touch*. Reports from earlier physicians, history, and our examination all confirm that Kim has never and still does not sense any type of stimulation of the body or face, including the corneas. Her insensitivity stems from a lack of functioning afferents, myelinated and unmyelinated alike, from the skin. A nerve biopsy and nerve conduction tests conducted early on provide evidence that this functional loss stems from an anatomical lack of cutaneous afferents.

Kim perceived touch stimulation of her tongue and palate with middling spatial accuracy. She was unable to localize oral stimuli to a side but could localize them to *above* or *below*. She also showed a clear gag reflex following stimulation of the uvula. This gag reflex was repeatable, occurred at short latency, and accompanied by appropriately negative affect. Thus, Kim displays some reflexive, non-discriminative sensitivity to light touch stimulation within the oral cavity. Notwithstanding this residual oral sensation, Kim has never chewed.

Gustatory tests revealed Kim to be ageusic. She did not correctly name tastants at even chance levels, answering *no taste* to most stimuli. She also professed not knowing the meaning of taste words. Thus, Kim does not have a sense of taste that at all resembles that of controls. On the other hand, she may perceive gustatory stimuli in a non-discriminative or protopathic manner. On occasion, she reacted to a tastant with an emphatic verbal report that was accompanied by congruent facial and verbal reactions. These instances were not consistently elicited by a particular taste or by higher concentrations of tastants, more evidence for protopathic rather than discriminative, intensity-graded, gustatory perception. Kim’s protopathic gustatory perception is consistent with her ageusic classification as psychophysical tests are designed to test solely for discriminative or epicritic perception.

Tests revealed Kim to be hyposmic. Kim scored above chance on two independent smell-identification tests, evidence that Kim detects, recognizes, and correctly names at least some specific smells. Smell is notoriously poorly coded in many languages including English (Majid et al 2014, 2018). Linguistic limitations include naming smells for their source (e.g. lemon) and the lack of abstract quality descriptors for odorants. Regardless, testing established that Kim’s deficits in olfactory perception are not of the same severity as those in cutaneous somatosensation and gustation.

In sum, Kim lacks sensitivity to somatic cutaneous and gustatory stimuli, which represent two of the three sensory modalities (olfaction being the third) most responsible for maternal-neonate bonding in mammals (Lipsitt 1977). Most importantly, she does not feel warmth, pressure, or taction from skin-to-skin contact or caressing strokes. She does not taste salt, sweet, bitter, or sour. In contrast, she can discriminate between odors, albeit less well compared to controls. She has normal hearing, vision, and vestibular function.

### Socioemotional and intellectual development

Neuropsychological testing revealed Kim to be a resilient and intellectually above-average woman with intact socioemotional processing. No social functioning deficits, emotional dysregulation, or rigidity, symptoms associated with ASD, were detected. Kim identified and described her own emotions while also recognizing others’ emotions. Thus, despite never experiencing touch, warmth, and taste, Kim developed normally in cognitive and socioemotional spheres.

Kim does not sense the signs of elevated sympathetic tone that mark anxiety. In contrast, she scored in the moderate to severe range for the affective and cognitive components of anxiety. Indeed, Kim reported episodes of heightened anxiety. Yet, as illustrated by her fear of the dying from not sensing a burst appendix, at least a portion of Kim’s fears are grounded in reality. As such, they likely engender an adaptive vigilance that serves Kim well as she navigates life without somatosensation. We interpret this vigilance as an appropriate *cultural* reaction to a pathological sensorium. In support of this interpretation, congenitally blind mice show less anxiety-like behavior and explore more (a sign of lower anxiety) than sighted controls (Bouguiyoud et al 2022). As further evidence against a biological vulnerability inherent to sensory loss, the subset of children with non-syndromic congenital deafness who develop anxiety are those who are challenged to communicate with hearing family members and peers (Wright 2008).

Kim gives long, detailed responses, even to simple questions, possibly as an offensive maneuver to broadcast her verbal skills, vocabulary, and intelligence (Infante and Wigley 1986). This strategy aimed at influencing the listener’s conception of Kim is likely motivated by extensive experience with strangers acting toward her as though she is cognitively impaired. Instances when she has been unrecognized as an intelligent, verbal person count as the most infuriating discriminatory acts that Kim has endured as a person in a wheelchair. Kim uses her most cooperative muscles – those of the larynx and upper airway – to preemptively counter the assumption that she is intellectually impaired. As further evidence of Kim’s strong desire to proactively counter strangers’ cognitive underestimation, Kim forcefully pushed for IQ testing which the lead author had rejected as insulting and unnecessary given Kim’s academic achievements. In line with Kim’s preference, IQ testing was conducted as reported above.

#### Touch is not required for socioemotional development

Kim’s healthy development even in the absence of touch can be attributed to two factors. First, touch is simply one component of many in the *maternal postpartum repertoire,* which in humans also involves carrying, moving, and bouncing (kinesthetic: somatosensory and vestibular); cooing, noises and speech (auditory), gaze and postural orientation (visual; Çetinçelik et al 2021, Field 2014, Akhtar and Gernsbacher 2008, Esposito et al 2013). Different combinations of caring repertoire components mark different cultural norms and yet all lead to normal infant development (Stack 2001, Akhtar and Gernsbacher 2008). For example, mothers in traditional societies make skin-to-skin contact with a child facing outward (*kangaroo care*) that promotes kinesthetic and passive touch stimulation throughout the day and night. In more individualistic societies, mothers hold their babies facing inward, thus emphasizing mutual gaze and active touch. While cultural differences convey cultural values to the infant, there is no evidence that only one particular admixture of maternal care components leads to healthy socioemotional development.

Additional evidence for flexibility in the caring repertoire comes from congenitally deaf or blind babies who grow up to be emotionally healthy adults (Hindley 2005, Demir et al 2014, Wright 2008). It is even possible for deafblind individuals such as Haben Girma to function socially and emotionally (Girma 2019). Kim provides solo but conclusive evidence that touch, pain, and taste are no more necessary for socioemotional development than are vision and hearing. The redundancy and flexibility in the effective maternal repertoire are not surprising given the consequential nature of social and affective function to mammalian survival.

The second factor that mitigates the adverse effects of a restricted caring repertoire is that any missing modality in the baby still operates in the mother (Harlow 1959, Bigelow and Power 2020). The care repertoire serves to bind the mother to the baby as well as the baby to the mother. In Kim’s case, her parents instantly loved her and fought for her care against dire medical predictions and cynical advice. As counterpoint, negative parental feelings about disability are the leading risk factor for emotional and behavioral problems among children with non-syndromic blindness or deafness (Hindley 2005, Wright 2008).

In sum, touch plays a role similar to that of vision and hearing in promoting socioemotional development. Each sense is integral to healthy socioemotional development when present, but no sense’s absence precludes healthy attachment of the infant to the caregiver. Nor does a child’s sensory deficit have an obligatory effect on the caregiver’s attachment to the child.

#### The importance of love

The gestalt of sensory input involving positive attention oriented from mother to offspring is read as *care* and drives the infant’s socioemotional development. Another term for what passes between caregiver and infant is *love* (Zeki 2007, Blumenthal and Young 2023, Harlow 1958). While *love* was the term used by Harry Harlow for caring behavior (Harlow 1958), it has largely disappeared from the biomedical lexicon (*love* + *review* yields 2,208 references), replaced by the operational term *attachment* (*attachment* + *review* yields 24,863 references). Here, we argue that *love* is the critical biological engine that fuels the affective bond between mother and child. In the words of Zeki (2007), it serves to “[overcome] social distance by deactivating networks used for critical social assessment and negative emotions, while it bonds individuals through the involvement of the reward circuitry, explaining the power of love to motivate…”

In current biomedical literature, the term *love* is used exclusively in reference to humans. Yet, love has its roots in attachment, evident in other mammals (Zeki 2007, Blumenthal and Young 2023). Restricting the word love to humans smacks of human exceptionalism and appears unwarranted. Physical closeness is requisite to the care of mammalian young as is blind tolerance of a young offspring’s oftentimes difficult behavior. The goals of reducing obstacles to physical closeness and minimizing affective reserve toward one’s young are absolute requirements for survival. Maternal love is an affective force that accomplishes both goals. Distinguishing between the affective state of love in humans and that of attachment in non-humans is unsupported by evidence.

## Conclusions

Kim’s condition is a natural experiment that excises touch while leaving vision, hearing, and vestibular senses intact. From studying her, we can conclude that touch *per se* is not necessary for a child’s socioemotional development. To say that touch is not necessary is *not* to say that touch cannot or does not influence the physical, social, and affective development of mammals under most circumstances. It does. Touch serves as a constitutive proxy for maternal care in everyone but Kim.

It is often repeated that somatosensation is the earliest sense to develop. In this vein, it has been argued that *in utero* taction allows the fetus to feel the effects of its own movements and those of its mother (Quintero and De Jaegher 2020). These tactile experiences when a fetal limb touches another part of the fetus or a part of the environment (the mother in this case) comprise the first “body feeling,” perhaps the kernel from which a *body schema*, the unconscious sense of oneself within the environment, will ultimately develop (Asai et al 2016, Quintero and De Jaegher 2020). Distinction between fetus-initiated and mother-initiated stimulation is an impetus for the “mother and fetus … pulling apart from each other”, thereby contributing to the fetus’s individuation (Quintero and De Jaegher 2020). Nonetheless, Kim, born insensitive to touch, emerged as a healthy and well-adjusted individuated woman with a strong sense of embodiment and self.

In conclusion, Kim tells us that there is considerably more to healthy affective development than touch. The key element to development is not the *means* of any one or combination of several sensory modalities. Rather it is the *end*, an ineffable feeling of being the target of attention and positive effect, that thing called *love*. The means are varied. The end is love.

## Data Availability

The data that support the findings of this study are available from the corresponding author, upon reasonable request.
